# *In Host* Mutational Adaptation of *Mycobacterium Tuberculosis* Complex Strains During Tuberculosis Infection

**DOI:** 10.1093/infdis/jiag209

**Published:** 2026-04-28

**Authors:** Helen Zhang, Naomi Medina-Jaudes, Alicia Forcada-Nadal, Ewan M Harrison, Francesc Coll

**Affiliations:** London School of Hygiene and Tropical Medicine, London, United Kingdom; London School of Hygiene and Tropical Medicine, London, United Kingdom; Applied Microbial Genomics Unit, Department of Molecular Basis of Disease, Institute of Biomedicine of Valencia (IBV-CSIC), Valencia, Spain; Parasites and Microbes, Wellcome Sanger Institute, Wellcome Genome Campus, Hinxton, Cambridge, United Kingdom; Department of Medicine, University of Cambridge, Cambridge, United Kingdom; Department of Public Health and Primary Care, University of Cambridge, Cambridge, United Kingdom; Applied Microbial Genomics Unit, Department of Molecular Basis of Disease, Institute of Biomedicine of Valencia (IBV-CSIC), Valencia, Spain

**Keywords:** *Mycobacterium tuberculosis*, bacterial evolution and adaptation, drug resistance, bacterial genomics, whole-genome sequencing

## Abstract

**Background:**

Tuberculosis (TB) is the leading cause of death from an infectious disease worldwide. Mutations arising in *Mycobacterium tuberculosis* complex (*MTBC*) strains during TB infection provide a record of bacterial adaptations, such as those needed to survive the attack of the immune system and antibiotic therapies.

**Methods:**

We conducted a meta-analysis of *MTBC* sequenced strains with multiple isolates from the same patient, sourced from published studies and TB Portals. We applied a convergent evolution approach to identify heavily mutated *MTBC* loci across patients and estimated the rates of drug resistance (DR) acquisition during treatment.

**Results:**

Using 5882 high-quality genomes from 1044 patients with TB, we identified 21 genes, 25 operons, and 27 promoter regions statistically enriched by mutations compared with the rest of the genome, and additional loci with established and plausible adaptive roles approaching statistical significance. Significantly, these included multiple loci known to be involved in resistance to first-, second-, and last-line anti-TB drugs. Previously reported candidate drug-resistance and -tolerance genes (*prpR*, *Rv2571c*, *fadD11*, *helY*, *ndhA*, *Rv0139*, *fadE5, bioF2*, and *mce1* operon) were also identified. Genes encoding regulators (*phoR, whiB6*, and *mycP_1_*) and effectors (*espK* and *eccE_1_*) of the virulence ESX-1 locus were frequently mutated *in host*. Fluoroquinolone resistance was acquired more frequently during treatment than resistance to any other anti-TB drug.

**Conclusions:**

We show that frequently mutated genes in *MTBC* reveal expected and newly discovered *in host* adaptations, predominantly associated with DR but also with pathogenesis. The higher resistance acquisition rate observed for fluoroquinolones may have important clinical relevance.

## Teaser

The authors applied a within-host evolutionary approach to show that frequently mutated genes in *Mycobacterium tuberculosis* complex strains reveal expected and newly discovered *in host* adaptations, predominantly related to drug resistance but also in genes of the virulence ESX-1 locus.

The application of evolutionary and functional genomic approaches have accelerated the discovery of novel drug resistance (DR) genes in *M. tuberculosis complex* (MTBC), the causative pathogen of Tuberculosis (TB), beyond canonical DR loci [1]. Genome-wide association studies (GWAS) of drug-resistant and -sensitive strains uncovered new loci associated with DR [2–8], some confirmed experimentally to mediate drug tolerance or resistance in vitro [2, 3, 8]. Genome-wide evolutionary approaches, such as those based on metrics of selection [9] or phylogenetic convergence [10], have also been successful at revealing candidate loci relevant for host–pathogen interactions and DR.

Previous studies investigated *MTBC* within-host diversity using whole-genome sequencing, most often to distinguish relapse versus re-infection in cases of recurrent TB, to quantify the degree of within-host diversity, to study DR evolution (reviewed here [11]), and to enhance the resolution of transmission inferences in genomic epidemiology studies [12, 13]. However, fewer studies have leveraged within-host diversity to study *in host* mutational adaptation [14–16]. A previous study [16] examined 200 patients with persistent or relapsed clonal infections and found 7 known DR genes and 3 genes of unknown function under parallel evolution. Another study analyzed a dataset of over 51 000 *MTBC* clinical isolates, quantified heterozygous mutations as a surrogate for within-host diversity, and identified known DR genes and transcriptional regulators repeatedly mutated across multiple patients [14].

Among comparative and population-based studies, within-host evolution studies have been particularly successful at identifying mutational adaptations in bacteria [16–18]. The power to detect signals of convergent evolution is high when clonal genomes from the same strain and host are compared, and if large number of strains/hosts are considered. The mutations fixed in the genomes of clinical strains provide a record of bacterial diversification and adaptation *in host*, and indirect evidence of the selective pressures bacteria encounter in vivo. Because *MTBC* is an obligate human pathogen [19], all selective forces acting on *MTBC* are expected to originate within the host, including those exerted by antibiotic therapies, nutritional limitations of the host and the immune system.

Here, we analyzed a dataset of 5800 *MTBC* genomes from more than 1000 patients with TB sourced from published studies and applied a genome-wide mutation enrichment approach to identify mutational adaptations. Overall, we identified 21 genes, 25 operons, and 27 promoter regions statistically enriched by mutations and more loci with established or plausible adaptive roles approaching statistical significance. In addition to canonical DR loci, we found candidate DR loci identified elsewhere by independent GWAS and functional genomics studies. We found recurrent mutations in loci regulating ESX-1 secretion transcriptionally (*phoR, whiB6*) and post-transcriptionally (*mycP1*), and in effector ESX-1 encoded proteins (*espK* and *eccE_1_*). Notably, *gyrA* was the most mutated DR locus, attributable to a higher de novo acquisition rate of fluoroquinolone resistance.

## METHODS

### Data Sources

We aimed to identify published collections of multiple *M. tuberculosis* complex (MTBC) isolates sequenced from the same patient with TB, whether collected longitudinally or from the same specimen (same day), from the NCBI Short Read Archive and TB Portals [20] (https://tbportals.niaid.nih.gov/), both queried on April 2022. We also extracted MTBC genomes and metadata from patients in the same studies who had a single MTBC isolate sequenced per host as contextual isolates (see Supplementary Methods for details on selection criteria).

### Genomic Analyses Applied to All MTBC Genomes

Kraken v2.1.2 [21] was used with default parameters and database (*kraken2-build –standard*) from raw fastq files to identify non-MTBC contamination. TB-Profiler v4.2.0 [22] (https://github.com/jodyphelan/TBProfiler) was used to assign MTBC lineage and sublineages and identify DR mutations. Illumina short reads were mapped to an ancestral version of the *M. tuberculosis* H37Rv reference genome [23] with Snippy v4.6.0 (https://github.com/tseemann/snippy) to call SNPs and short indels (consensus calls). Reads were assembled using a computational pipeline based on SPAdes assembler [24] v3.15.3 and the improve_assembly pipeline (https://github.com/francesccoll/assembly_pipeline). Illumina single-end reads were assembled using SKESA 2.4.0 [25]. The quality of all assemblies was evaluated using QUAST v5.0.1 [26]. See genomic QC metrics and thresholds applied in Supplementary Methods.

### Genomic Analyses Applied to Isolates of the Same Host

To avoid comparing the genomes of divergent strains from the same individual (ie, mixed infections), only clonal isolates were kept for further analyses. Clonality was ruled out if isolates belonged to different TB-Profiler sublineages or to the same sublineage separated by more than 10 SNPs, a stringent genetic relatedness distance equivalent to the previously established cutoff for *M. tuberculosis* cross-transmission [27]. We applied a previously developed pipeline [18] to identify de novo genetic variants originating during infection from multiple isolates sequenced from the same host (see Supplementary Methods). Variants in repetitive regions, regions of low complexity, and hard-to-genotype [28] were excluded from further analysis, overall 0.74 Mb out of 4.41 Mb in the H37Rv reference genome (16.7%). The final set of high-quality variants were annotated using *SnpEff* v4.3 [29] in the H37Rv reference genome.

### Genome-wide Mutation Enrichment Analysis

A convergent evolution approach was applied to identify heavily mutated loci across multiple patients [18]. We counted protein-altering mutations (ie, those having HIGH or MODERATE annotation impact by *SnpEff*) within protein coding sequence and operons, and all mutations within RNA coding and intergenic region. For each patient, we deduplicated de novo mutations detected in multiple *MTBC* genomes of the same strain to ensure unique genetic changes were counted only once (ie, assumed to have originated once in host). We additionally considered mutations within 100-bp upstream of the transcription start site of each operon, which are expected to contain promoter and regulatory sequences. The *M. tuberculosis* H37Rv annotated genome (release 4, 2021-03-23) was downloaded from https://mycobrowser.epfl.ch/releases, and transcriptional units (operons) from https://mycobacterium.biocyc.org/. We tested CDS and operons for an excess of protein-altering (functional) mutations compared with the rest of the genome, considering the length of CDS, or cumulative length of CDS if testing operons involving multiple CDS. We performed a single-tailed Poisson test using the genome-wide mutation count per bp multiplied by the gene length as the expected number of mutations. *P* values were corrected for multiple testing using a Benjamini & Hochberg correction. We chose a significance level of .05 and reported hits with an adjusted *P* value below this value, unless otherwise stated.

#### Determination of Drug-resistance Acquisition Rates

Anti-TB treatment data were extracted from TB Portals and published studies when available (Supplementary Table 3). Binary variables were created for each drug to indicate whether each patient had received that drug or not over the course of treatment. The number of strains that had a fixed mutation on any of the genes known to be associated with DR (Supplementary Table 2) were tallied. The DR acquisition rate was calculated as the number of strains treated with a drug and had a fixed de novo mutation on any DR gene divided by the number of strains treated with that drug. For each proportion, a 95% CI was calculated. Multivariate logistic regression models, implemented with glm(family = binomial) in R version 4.5.2, were used to test the effect of co-variates—treatment with fluoroquinoles, time between isolate collection, and baseline genotypic DR (pan-susceptible vs any form of DR)—on the odds of developing fluoroquinolone resistance mutations.

## RESULTS

### Characteristics of the Compiled Dataset of MTBC Genomes

**Figure 1.**
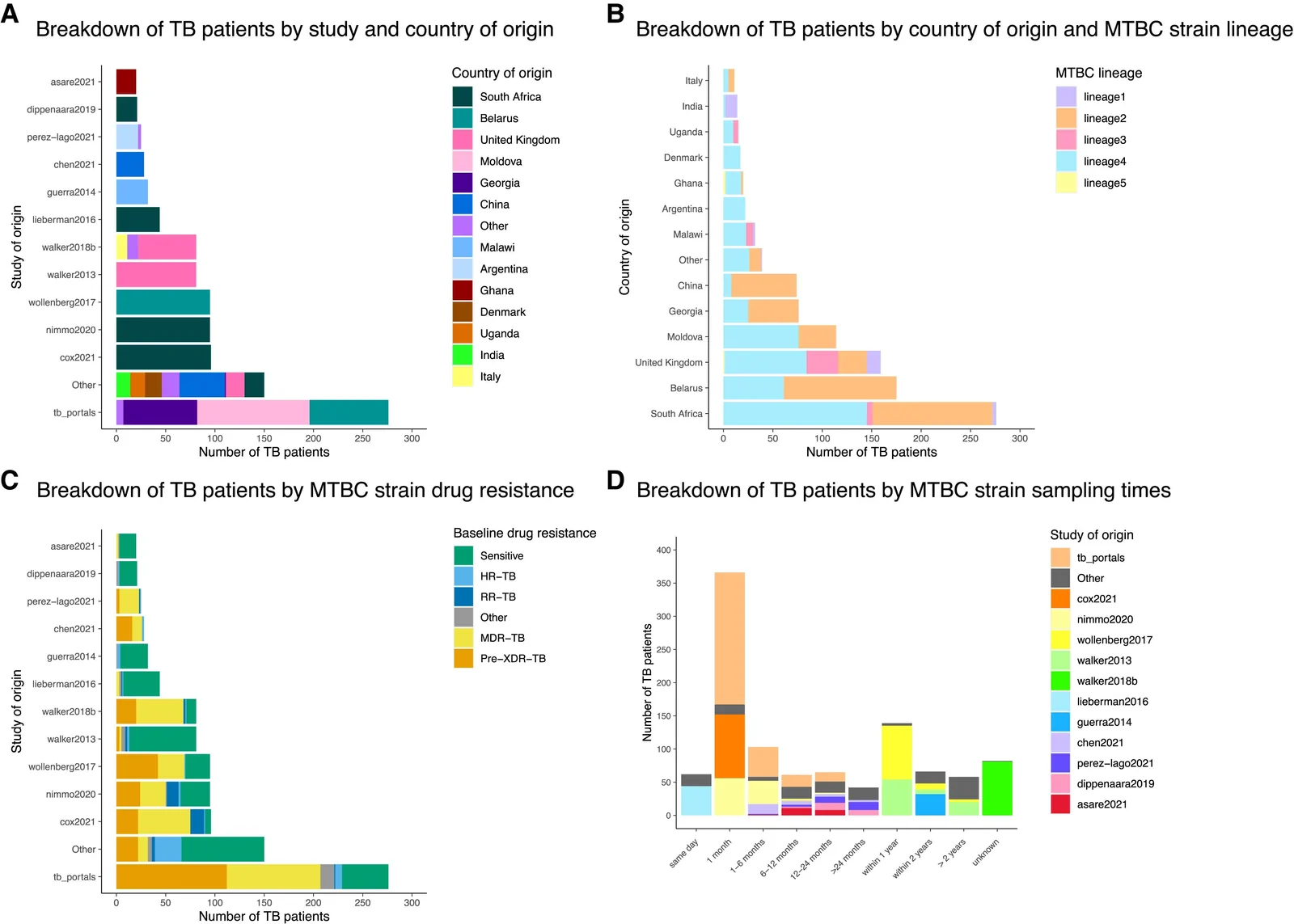
Characteristics of compiled MTBC strain dataset used in this study. *A,* Breakdown of patients with TB by study and country of origin. The share of patients with TB by study is color-coded by country of origin. Studies contributing less than 20 patients with TB and countries contributing less than 10 patients are labeled as “Other”. *B*, Breakdown of patients with TB by country of origin and MTBC strain. The share of patients with TB by country of origin is color-coded by MTBC lineage, as determined from genome sequences by TB-Profiler. *C*, Breakdown of patients with TB by the baseline DR of their MTBC strain. The share of patients with TB by study is color-coded by baseline genotypic DR, as determined from genome sequences by TB-Profiler. Baseline DR is defined considering all isolates genomes from the same strain and host, and assuming the least DR patter is that of the original (baseline) infecting strain. *D*, Breakdown of patients with TB by MTBC sampling times. The maximum time distances between sample collection times of all isolates from the same strain and host are shown in the x-axis. The share of patients with TB by collection time distances is color-coded by study.

A total of 7011 *MTBC* isolate genomes were identified from the NCBI and TB Portals (n = 856) [20], from 34 different studies (n = 6155) (Supplementary Table 1)—which originated from 1409 different patients with TB (median of 2 isolates sequenced per host, 2-3 IQR). An additional 5518 isolates from other patients in the same studies were considered as contextual isolates (Supplementary Data 1 and Supplementary Table 1). After genomic QC, 5882 *MTBC* genomes (81.8%) from 1044 hosts (70.8%) were kept (Figure 1*A*), plus an additional 5417 contextual isolates.

*MTBC* strains originated from 27 different countries (Figure 1*A*), mostly sampled from Europe (55.4%) and Africa (32.9%). As expected, most *MTBC* strains belonged to lineage 4 (49.7%) and 2 (42.0%). Most patients had isolates grown from pulmonary specimens (55.4%), most often from sputum specimens only (526, 50.4%). The anatomical origin of samples was not reported for 408 patients (39.1%). In terms of baseline genotypic DR, there was an even representation of pan-susceptible (35.4%), MDR (28.4%), and pre-XDR strains (25.3%) (Figure 1*C*).

### Genome-wide Mutation Enrichment Analysis Identifies Well-known Drug Resistance Loci

**Figure 2.**
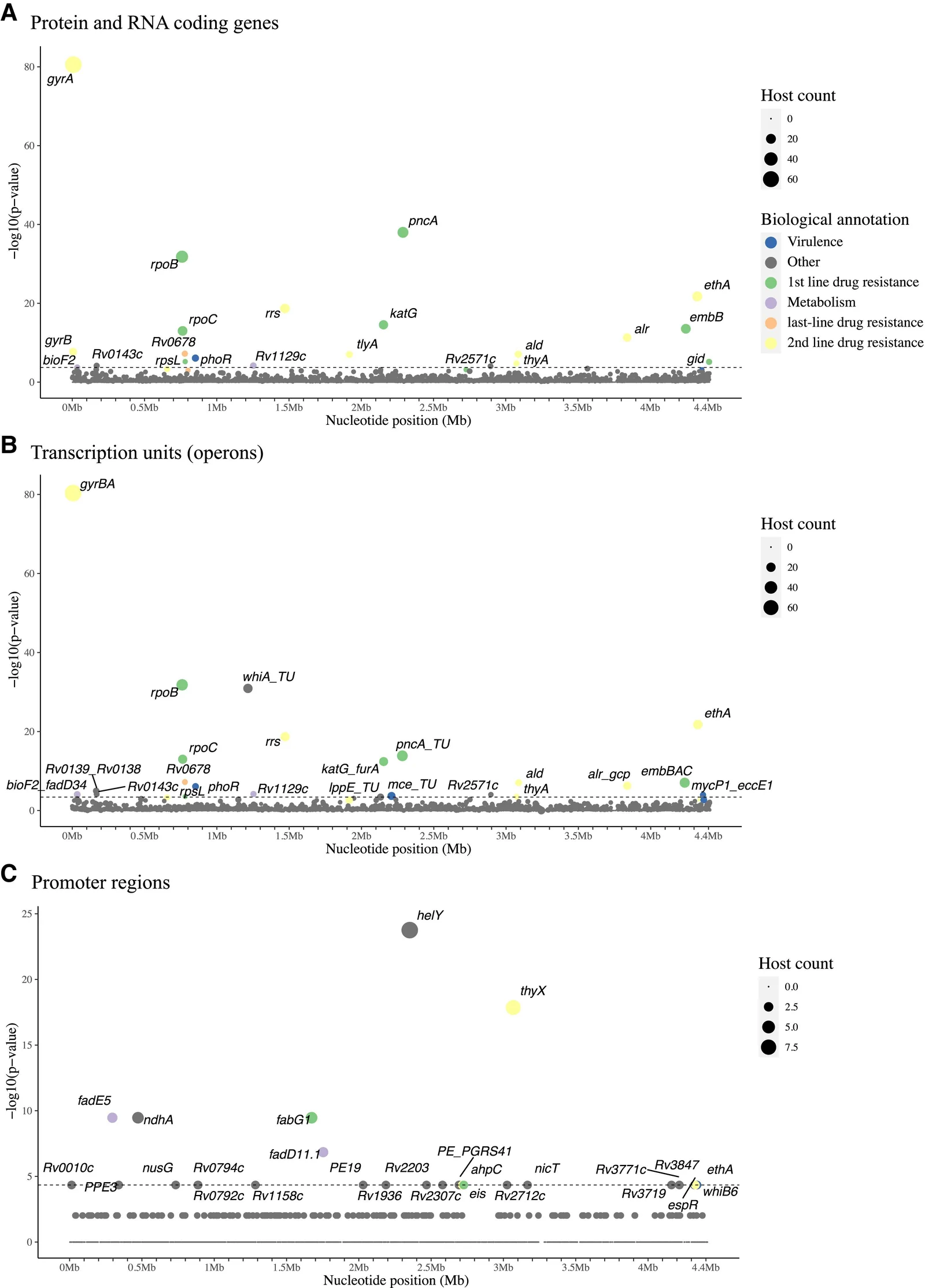
Loci enriched with mutations across multiple MTBC strains. *A*, Protein and RNA coding genes, (*B*) transcriptional units (operons), and (*C*) promoter regions (100 bp upstream of annotated operons) enriched for mutations across the MTBC strains of multiple TB patients. Each circle denotes a single locus, whose size is proportional to the number of hosts mutations arose independently from. Loci are placed at the x-axis based on their chromosome coordinates. Loci are color-coded by biological functions, particularly if they are known to be involved in DR or virulence. The y-axis shows the uncorrected *P*-value resulting from a single-tailed Poisson test comparing the density of mutations per functional unit against the expected number of mutations, obtained from multiplying the genome-wide mutation count per bp by the gene length (see Methods). The dotted horizontal line represents the genome-wide statistical significance threshold. Mutations identified in a collection of 5882 *MTBC* isolate genomes from 1044 individuals (see Supplementary Table 1).

Using clonal *MTBC* isolates from the same host, we identified 3296 fixed mutations across 503 strains (out of 1060 strains from 1044 patients) that arose de novo during TB infections, with a median of 2 mutations per strain (1–5 IQR). We applied a genome-wide mutation enrichment approach to identify loci in the *MTBC* genome exhibiting evidence of adaptation, as previously implemented [18]. We found 21 genes, 25 operons, and 27 promoter regions statistically enriched by mutations compared with the rest of the genome (Figure 2). We observed 338 mutations in these 21 genes (34 Kb, 9.28 × 10^−6^ mutations per site) compared with the 3296 mutations across the whole genome (3675 Mb, 8.46 × 10^−7^), a 10.9-fold higher than the background genome-wide mutation rate (95% CI: 9.8–12.3, Poisson test *P*-value < 2.2 × 10^−16^). Among associated operons, we found a similar mutation rate (77 Kb, 4.82 × 10^−6^ mutations per site), 5.7-fold higher than the background rate (95% CI: 5.1–6.3, *P*-value < 2.2 × 10^−16^); and among associated promoter regions (2.7 Kb, 1.31 × 10^−5^ mutations per base), a 15.5-fold higher rate (95% CI: 10.9–21.4, *P*-value < 2.2 × 10^−16^).

As expected, many loci with signals of *in host* mutational adaptation are known DR loci (Supplementary Table 2). We identified loci linked to resistance to first-line drugs (*katG*, *ahpC* promoter, *inhA* promoter, *rpoB*, *rpoC, pncA,* and *embB*), second-line drugs (*gid, rpsL, rrs, eis* promoter, *tlyA, gyrA*, *gyrB*, *ethA,* and *ethA* promoter), and last-line drugs (*alr*, *ald, Rv0678, thyA,* and *thyX* promoter) (see Supplementary Table 2 for corresponding drugs). Other well-characterized DR loci were found approaching statistical significance (n = 7), including loci involved in resistance to isoniazid (*inhA, ahpC, Rv2752c,* and *ndh*), ethionamide (*Rv0565c* and *inhA*), and linezolid (*rplC*). Notably, among all DR genes, *gyrA* was by far the most mutated locus. Next, we compared individual mutations in these DR loci against those in the WHO catalogue of DR mutations [1], except for cycloserine (*ald* and *alr*) and para-aminosalicylic acid (*thyA* and *thyX* promoter) loci, which are not included in the catalogue. We found 323 independently acquired mutations in 164 different strains (218 unique variants) across 31 DR loci (Supplementary Data 2). Out of these 218 unique variants, 35 (16%) are not listed in the WHO catalogue, 93 (42.7%) are reported to be associated with resistance, 7 (3.2%) not associated, and 83 (38.1%) associated with uncertain significance.

### *In Host* Mutational Adaptation Reveals Less Characterized and Candidate Drug Resistance Loci

The genes *prpR* (*Rv1129c*), *Rv0143c*, *Rv2571c,* and *bioF2 (Rv0032)* were statistically enriched by mutations *in host*, as many canonical DR genes were (Figure 2*A*). These genes had an average mutation rate 4.9-fold higher (4.18 × 10^−6^ mutations per site) than the background mutation rate (95% CI: 3.3–7.1, Poisson test *P*-value < 1.92 × 10^−11^) but 0.4-fold lower than that in canonical DR genes (95% CI: 0.26–0.58, *P*-value < 1.14 × 10^−7^). Mutations in *prpR*, a regulator of propionate metabolism, have been reported to confer tolerance to multiple drug classes [2]. In a GWAS of MDR-TB strains [5], *Rv2571c* was found associated with ethambutol resistance, and mutations in *bioF2* were proposed to be implicated in streptomycin resistance [30]. No role in DR is reported for *Rv0143c*, a gene encoding a putative transmembrane chloride channel transporter.

When considering candidate promoters, the promoter region of *helY* (*Rv2092c*) was the most mutated promoter region (Figure 2*C*). *helY* promoter mutations might be involved in DR as its coding region (encoding the DNA helicase HelY) was reported to be associated with isoniazid resistance [2]. We found an enrichment of mutations in the *ndhA* promoter, consistent with previous observations that mutations in *ndhA* (*Rv0392c*, encoding an NADH dehydrogenase) are phylogenetically associated with resistance and that *ndhA* transposon knockouts exhibit increased isoniazid resistance [31]. We also found *Rv0139* (encoding a possible oxidoreductase) approaching statistical significance, also linked to isoniazid resistance by transposon mutagenesis [31], and consistent with the role of redox metabolism in isoniazid resistance [32].

We additionally identified the promoters of *fadE5* (*Rv0244c*) and *fadD11-plsB1* (*Rv1550- Rv1551*) with evidence of convergent evolution (Figure 2*C*). Overexpression of *fadE5* in *M. smegmatis*, encoding an acyl-CoA dehydrogenase, has been shown to increase resistance to ethambutol and streptomycin [33]; while knockout mutants of *fadE5* in *MTBC* exhibit reduced tolerance to rifampicin [34]. A recent GWAS found loss-of-function mutations in *fadD11* associated with streptomycin resistance [4]. Among operons, the *mce1* operon (*Rv1964* to *Rv1975*) was among the top mutated ones (Supplementary Data 3) (Figure 2*B*). Transposon knockouts of *mce1* operon genes display increased isoniazid resistance [31], consistent with their role in cell wall lipid transport and homeostasis [35].

### Loci Encoding Regulators of Virulence-associated ESX-1 Locus are Often Mutated in Host

**Figure 3.**
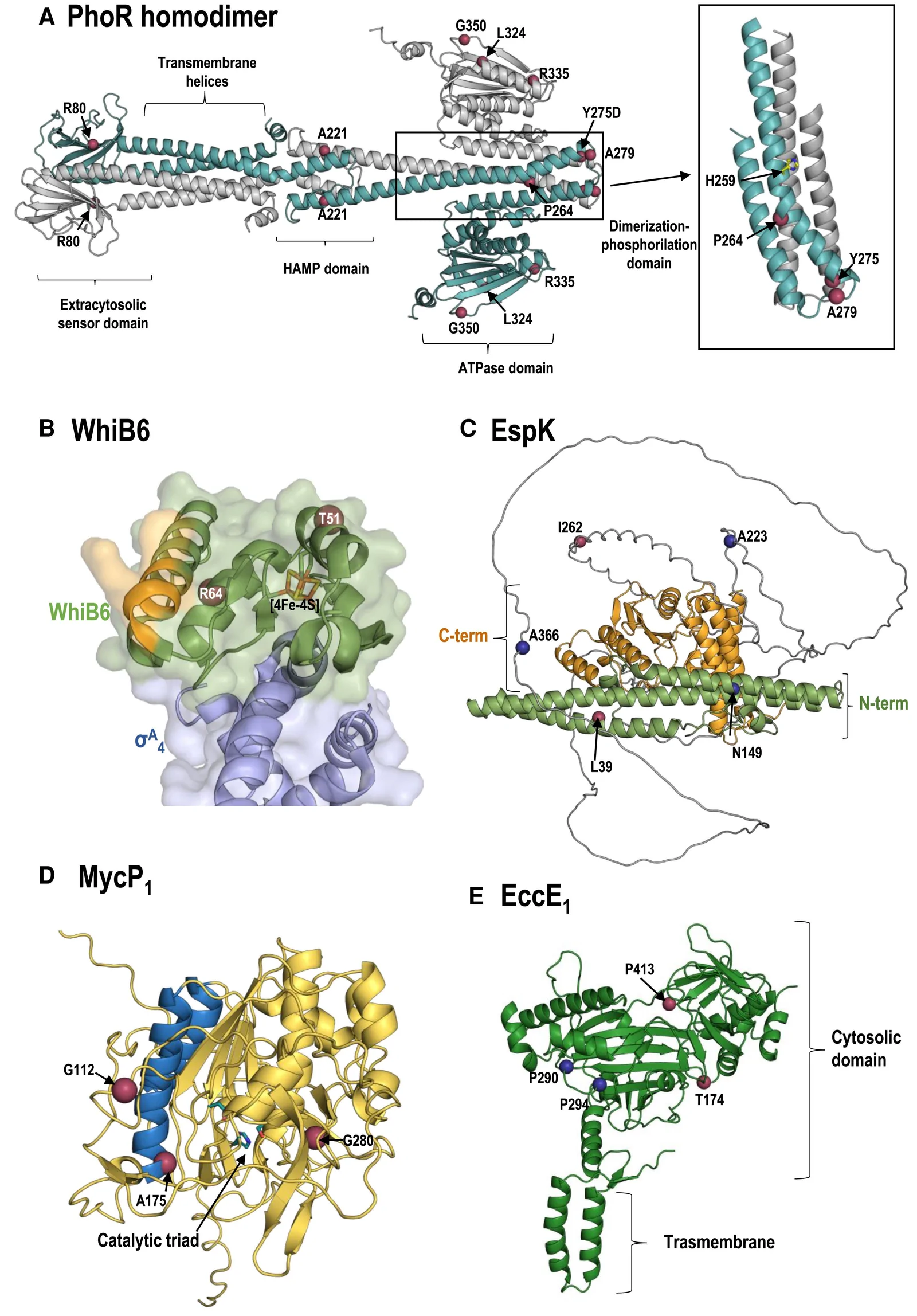
Localization of the protein structure of mutations in ESX-1-associated proteins. *A*, Structural model of the *M. tuberculosis* PhoR homodimer predicted by AlphaFold. One subunit is shown in green, and the other in gray. Mutations identified in this study are highlighted as red spheres. The inset shows the dimerization-phosphorylation domain from the crystal structure (PDB 5UKV), with mutation positions indicated in a single subunit for clarity. The active-site His259 residue involved in autophosphorylation is shown as sticks. *B*, Structure of the *M. tuberculosis* WhiB6 (green)–σA4 (blue) complex (PDB 8DV5). The identified mutations are shown as red spheres. The [4Fe–4S] cluster and its coordinating cysteine residues are represented as sticks, and residues predicted to interact with DNA are highlighted in yellow. *C*, AlphaFold model of *M. tuberculosis* EspK. The N-terminal domain is shown in green, the C-terminal domain in yellow, and the long flexible linker in gray. Point mutations are represented as red spheres and frameshift mutations as blue spheres. *D*, AlphaFold model of *M. tuberculosis* MycP1. Mutated residues are shown as red spheres. The catalytic triad is represented as green sticks, and the helices involved in substrate binding are highlighted in blue. The transmembrane helix is omitted for clarity, as no mutations were located in this region. *E*, AlphaFold model of *M. tuberculosis* EccE1. Residues bearing point mutations are shown as red spheres and those with frameshift mutations as blue spheres.

The most frequently mutated gene *in host* beyond canonical DR genes was *phoR*, the gene encoding the sensor kinase of the *phoPR* 2 component system, which controls the secretion of ESAT-6, considered one of the major virulence factors of *MTBC*. Consistent with this finding, previous genomic studies have found strong evidence of positive selection in *phoR* [9]. We identified 8 PhoR mutations (Figure 3*A*), particularly in the dimerization-phosphorylation and ATPase domains, including 6 mutations that would change the electrostatic surface potential (Supplementary Figure 3) or compromise the protein's structural stability (Supplementary Table 4) (see Supplementary Results for details).

Functionally related to PhoR, we found an enrichment of mutations in WhiB6 (*Rv3862c*) and in the ESX-1 secretion-associated protein EspK (*Rv3879c*), both approaching statistical significance (Supplementary Data 3). *whiB6* is located adjacent to the ESX-1 locus and is a known ESX-1 transcriptional regulator. The expression of *whiB6* is positively regulated by PhoP, which binds directly to its promoter [36]. Figure 3*B* shows that WhiB6 substitutions (Thr51Pro, detected twice, and Arg64His), both predicted to be destabilizing, are located close to the conserved cysteines coordinating the [4Fe-4S] cluster. Interestingly, a recent study [37] found 13 independent WhiB6 variants linked to decreases in PE35-PPE68-esxBA operon expression, including substitutions close to the ones detected here (Cys53Gly, Asp46Ala). We identified 5 mutations from 4 different patients in EspK (Figure 3*C*), an auxiliary protein that recruits ESX-1 substrate EspB [38], including 3 frameshifts and one strongly destabilizing missense mutation, which would compromise EspB secretion.

When considering operons, the one encoding MycP_1_ (*Rv3883c*) and EccE_1_ (*Rv3882c*) was statistically enriched by mutations. MycP_1_ is a serine protease that regulates substrates of ESX-1 like EspB post-transcriptionally [39], and EccE_1_ an effector membrane component of ESX-1 required for secretion of ESX-1 substrates [40]. In MycP_1_, we identified 3 missense mutations (Figure 3*D*), including 2 with moderate-to-strong destabilizing effects, which might influence substrate recognition or enzyme dynamics, and in EccE_1_ 4 mutations (Figure 3*E*) including 2 loss-of-function frameshift variants.

### Fluoroquinolone Resistance was Acquired More Frequently Than Resistance to any Other Drug

We observed that *gyrA* was the most frequently mutated gene (Figure 2*A*) and investigated whether a higher use of fluoroquinoles in treatment or a higher DR acquisition rate could explain this observation. Drug treatment data were available for 550 (52%) of all strains (n = 1066, Supplementary Table 3, Supplementary Figure 2). Strains were treated with a range of anti-TB drugs, most often with pyrazinamide (n = 438, 79.6%), fluoroquinolones (n = 352, 64.0%), and ethambutol (n = 298, 54.2%) (Table 1). Fluoroquinolone resistance was acquired more often than to other drugs: of the 352 strains treated with fluoroquinolones, 46 acquired a fixed DR mutation in *gyrA* or *gyrB* (13.1%, 95% CI 9.9%–17.0%, see Table 1), compared with only 2 strains among not treated strains (1.01%, 95% CI: 0.25%–3.99%), a statistically significant 14.7-fold change (95% CI: 3.8–126.5, Fisher's exact test *P*-value = 1.28 × 10^−7^). In a multivariate logistic regression model using treatment with fluoroquinoles, time between isolate collection, and baseline genotypic DR as covariates, only treatment with fluoroquinoles was associated with *gyrA/B* mutations (OR: 7.8, 95% CI: 1.6–38.6, *P*-value = .0113). The acquisition rate for fluoroquinolone resistance was higher among MDR strains (17.9%, 95% CI: 12.5%–25.1%).

**Table 1.** Frequency of Genotypic Drug Resistance Acquisition in MTBC Strains

	All Strains (n = 550)			Pan-susceptible Strains (n = 174)			MDR Strains (n = 158)			XDR Strains (n = 158)		
Drug	N DR^a^	Drug Used	Acquisition Rate (95% CI)	N DR^a^	Drug Used	Acquisition Rate (95% CI)	N DR^a^	Drug Used	Acquisition Rate (95% CI)	N DR^a^	Drug Used	Acquisition Rate (95% CI)
Amikacin	3	76	3.9% (1.2–11.8%)	1	3	33.3% (2.5–90.8)	0	35	0% (0–0%)	0	23	0% (0–0%)
Bedaquiline	4	165	2.4% (0.9–6.3%)	0	28	0% (0–0%)	0	50	0% (0–0%)	3	50	6.0% (1.9–17.3%)
Capreomycin	8	222	3.6% (1.8–7.1%)	1	5	20.0% (2.1–74.6%)	3	97	3.1% (1.0–9.3%)	2	91	2.2% (0.5–8.5%)
Clofazimine	5	144	3.5% (1.4–8.2%)	0	8	0% (0–0%)	0	49	0% (0–0%)	3	51	5.9% (1.9–17.0%)
Cycloserine	13	245	5.3% (3.1–9.0%)	0	6	0% (0–0%)	5	108	4.6% (1.9–10.7%)	6	97	6.2% (2.8–13.2%)
Ethambutol	8	298	2.7% (1.3–5.3%)	2	167	1.2% (0.3–4.7%)	2	67	3.0% (0.7–11.3%)	2	25	8.0% (1.9–27.7%)
Ethionamide^b^	11	258	4.3% (2.4–7.6%)	1	12	8.3% (1.0–43.8%)	3	111	2.7% (0.9–8.1%)	4	94	4.3% (1.6–10.9%)
Fluoroquinolones^c^	46	352	13.1% (9.9–17.0%)	0	33	0% (0–0%)	26	145	17.9% (12.5–25.1%)	13	144	11.4% (6.7–18.7%)
Isoniazid	8	231	3.5% (1.7–6.8%)	4	127	3.1% (1.2–8.2%)	1	46	2.2% (0.3–14.3%)	1	22	4.5% (0.6–27.3%)
Kanamycin	1	31	3.2% (0.4%–21.6%)	1	5	20.0% (1.9–76.3%)	0	11	0% (0–0%)	0	5	0% (0–0%)
Linezolid	2	121	1.7% (0.4%–6.5%)	0	1	0% (0–0%)	0	46	0% (0–0%)	1	51	2.0% (0.3–13.1%)
Pyrazinamide	16	438	3.7% (2.2%–5.9%)	1	169	0.6% (0.1–4.1%)	6	118	5.1% (2.3–10.9%)	7	84	8.3% (4.0–16.6%)
Rifampicin	3	184	1.6% (0.5%–5.0%)	1	138	0.7% (0.1–5.1%)	0	16	0% (0–0%)	1	4	25.0% (2.3–82.2%)
Streptomycin	2	39	5.1% (1.2–19.3%)	1	24	4.2% (0.5–26.4%)	0	4	0% (0–0%)	0	2	0% (0–0%)

Number of strains that acquired genotypic DR during treatment, as determined by protein-altering mutations in corresponding DR loci, in MTBC strains known to have been treated and exposed to the corresponding drug.

^a^Number of strains with at least one mutation in DR loci.

^b^Ethionamide includes prothionamide.

^c^Fluoroquinolones include treatment with levofloxacin, moxifloxacin, or ofloxacin.

## DISCUSSION

Here, we conducted a meta-analysis of multiple *MTBC* isolates sequenced from patients with TB to capture within-host genetic diversity and identify signals of *in host* adaptation. We used over 5800 high-quality *MTBC* genomes from more than 1000 patients with TB sourced from TB-Portals and published studies and of diverse phylogenetic and geographical origins. We identified mutations that arose de novo in *MTBC* strains during infection and identified loci with recurrent mutations, providing indirect evidence of the evolutionary pressures that *MTBC* encountered *in host*. Overall, we identified 21 genes, 25 operons, and 27 candidate promoter regions in the *MTBC* genome statistically enriched by mutations and more loci with an established or plausible adaptive role approaching statistical significance.

The identification of many canonical DR genes demonstrated the robustness of our approach. These confirmatory results give credibility to the putative adaptations identified in ESX-1 loci and candidate DR loci, identified in GWAS of clinical strains (*prpR*^2^, *Rv2571c* [5], *fadD11* [4] and *helY* [2]) or in transposon mutagenesis studies (*ndhA, Rv0139,* and *mce1* operon) [31]. Our results show that within-host evolutionary studies can complement GWAS and functional genomics approaches in identifying new DR candidate loci.

Interestingly, we found a higher acquisition rate of fluoroquinolone resistance compared with that of other drugs. Longitudinal sampling of *MTBC* showed that de novo acquisition of resistance is particularly high for fluoroquinolones [41]. Similar rates of acquired fluoroquinolone resistance were reported in studies of MDR TB (12.1% and 9.1%) [42, 43], although these studies reported higher acquired resistance rates to second-line injectable drugs (7.8% and 9.8%) than the ones detected here. The higher rate of *gyrA* mutations could be in part attributable to the common empirical use of fluoroquinolones to treat other community infections [44]. Higher rates of heteroresistance to fluoroquinolones [2, 45] may also explain the higher resistance acquisition rate measured here.

Beyond resistance to anti-TB drugs, our observations point to an important role of adaptive mutations in regulators and effectors of the ESX-1 secretion system, most often in *phoR* but also in other transcriptional (*whiB6*) and post-translational (*mycP_1_*) regulators and effectors (*espK* and *eccE_1_*) of this system. Mutations identified in these genes could therefore influence ESX-1 activity at multiple levels, from gene regulation to substrate recognition and secretion, and directly impact host–pathogen interactions. Previous studies have identified *phoR* as a target of positive selection [9], and a recent study [37] found repeated evolution of WhiB6 variants linked to decreased expression of ESX-1 effectors, expected to lead to attenuated virulence. The identification in our study of loss-of-function mutations in EspK and EccE_1_, both required for secretion of ESX-1 substrates, also points toward an adaptation toward lower virulence. Alternatively, genetic variants in ESX-1 regulators may allow *MTBC* cell populations to diversify *in host* and create subpopulations of cells preadapted to different conditions or immunological contexts (balancing selection), for example, in different lung cavities [46]. The recent identification of common phase variants upstream of other ESX-1 regulators [47] points to the importance of maintaining heterogeneity in ESX-1 expression *in host*. Future work should determine if factors influencing the host immune response (eg, prior immune exposure, BGC vaccination, age, nutritional status, or HIV status) [48] could influence selective pressures on ESX-1 and thus the presence and spectrum of adaptive mutations in this locus.

The diversity of mutational adaptations observed reflects the adaptability of *MTBC* strains and plasticity of their genome. *MTBC* evolves mostly through mutations due to errors in DNA replication. Indeed, defects in DNA repair genes that have been linked to mutator phenotypes and emergence of DR in *MTBC* [49]. Future studies should explore links between defects in DNA repair, mutation rates, and the extent of adaptive mutations. Population-based studies could determine the long-term evolutionary success of adaptive variants or determine if they are transient and short-lived instead.

Our study has several limitations. First, most *MTBC* strains had only 2 isolates sequenced per host, limiting the amount of genetic diversity captured from clinical specimens. Sequencing directly from sputum in large cohorts of patients with TB would provide more realistic estimates of the prevalence and acquisition rate of the adaptations identified here. Third, we only considered fixed genetic variants and excluded heterozygous variants, which can represent low abundant adaptive mutations on a trajectory of fixation [16], fixed at certain anatomical sites [46], or in balancing selection. Fourth, rather than identifying specific sites enriched for substitutions, we used loci (genes, promoters, and operons) as the unit of analysis, which was necessary to increase the statistical power to detect loci under selective pressures. Fifth, due to inherent limitations of short-read data, we had to hard-to-genotype regions (eg, repetitive, high-G + C) [28]. We excluded 16.7% of positions on the reference genome, which may have led to an underestimation of adaptive mutations. Future work employing long-read sequencing approaches will overcome this limitation. Finally, drug treatment data only included which drugs were used, and no details on the duration of exposure or changes of drugs administered, which may have impacted our estimations of DR acquisition.

Here, we revealed known and new mutational hotspots in the *MTBC* genome, in loci of established and plausible adaptive roles during infection. The less characterized loci now warrant experimental validation to confirm the environmental stressors that selected adaptive mutations, the underlying molecular mechanisms mediating adaptation, and their impact on disease progression.

## Supplementary Material

jiag209_Supplementary_Data

## Supplementary Data

Supplementary materials are available at *The Journal of Infectious Diseases* online (http://jid.oxfordjournals.org/). Supplementary materials consist of data provided by the author that are published to benefit the reader. The posted materials are not copyedited. The contents of all supplementary data are the sole responsibility of the authors. Questions or messages regarding errors should be addressed to the author.

## Notes

***Acknowledgments***. Data were obtained from the TB Portals (https://tbportals.niaid.nih.gov), which is an open-access TB data resource supported by the National Institute of Allergy and Infectious Diseases (NIAID) Office of Cyber Infrastructure and Computational Biology (OCICB) in Bethesda, MD. These data were collected and submitted by members of the TB Portals Consortium (https://tbportals.niaid.nih.gov/Partners). Investigators and other data contributors that originally submitted the data to the TB Portals did not participate in the design or analysis of this study.

***Author Contributions.*** Conceptualization: F. C.; Data curation: F. C., H. Z., and N. M. J.; Formal bioinformatic analysis: F. C., H. Z., and N. M. J.; Funding acquisition: F. C.; Investigation: F. C., H. Z., N. M. J., and A. F. N.; Bioinformatics methodology: F. C., H. Z., and N. M. J.; Laboratory methodology: not applicable; Project administration: not applicable; Resources: F. C. and E. M. H.; Supervision: F. C.; Validation: not applicable; Visualization: H. Z., N. M. J., and A. F. N.; Writing—original draft: F. C.; Writing—review and editing: all authors.

***Data availability statement***. The whole-genome sequences of the isolate collections used in this study are available on European Nucleotide Archive (ENA) under the accessions listed in Supplementary Data 1, which also includes isolate metadata. All scripts necessary to run the described analyses are available on GitHub (https://github.com/francesccoll/mtbc-adaptive-mutations/). The list of raw, filtered, and DR mutations identified to have been originated de novo during infection can be found in Supplementary Data 2. The full list of genes, operons and promoter regions enriched by mutations can be found in Supplementary Data 3. Supplementary Data 4 includes drug treatment data for individual strains.

***Financial support.*** This publication presents independent research supported in part by a Wellcome grant 204928/Z/16/Z. This work was also funded by the Spanish State Research Agency (AEI) project PID2023-152061NA-I00, MCIN/AEI/10.13039/501100011033 FEDER, UE. The views expressed in this publication are those of the author(s) and not necessarily those of the funders.
