# Supplementary material for: In Host Mutational Adaptation of Mycobacterium Tuberculosis Complex Strains During Tuberculosis Infection
Source: J Infect Dis. 2026 Apr 28;234(3):e623–33. doi: 10.1093/infdis/jiag209 (PMC13600426; doi:10.1093/infdis/jiag209)
Supplement: Supplementary file 1 [file jiag209_supplementary_data.zip › Mtb_mutational_adaptation.v1.1.supplementary.revised.clean.docx]

**Supplementary Materials**

***In host* mutational adaptation of *Mycobacterium tuberculosis* complex strains during Tuberculosis infection**

Helen Zhang, Naomi Medina-Jaudes, Alicia Forcada-Nadal, Ewan M Harrison, Francesc Coll

**Supplementary Methods**

**Supplementary Results**

**Supplementary Tables**

Supplementary Table 1. Number of MTBC isolate genomes and TB patients available per study before and after genomic QC.

Supplementary Table 2 *M. tuberculosis* genes known to confer resistance to anti-tuberculosis drugs.

Supplementary Table 3. Data sources and treatment data availability.

Supplementary Table 4. FoldX Stability Predictions and Electrostatic Alterations.

**Supplementary Figures**

Supplementary Figure 1 Visual representation of genomic QC metrics and thresholds.

Supplementary Figure 2. Descriptive characteristics of all strains with available drug treatment data.

Supplementary Figure 3. Electrostatic surface potential of PhoR wild-type and variant proteins.

Supplementary Figure 4. Electrostatic surface potential of WhiB6, EspK, MycP1 and Ecc1 wild-type and variant proteins.

**Supplementary files**

Supplementary Data 1. List of isolate genomes, accessions and metadata used in this study.

Supplementary Data 2. List of raw, filtered, and drug resistance mutations identified to have been originated *de novo* during infection.

Supplementary Data 3. List of genes, operons and promoter regions enriched by mutations.

Supplementary Data 4. Drug treatment data.

**Supplementary References**

**Supplementary Methods**

***Data sources: selection criteria and metadata extraction***

The NCBI Short Read Archive (SRA) was queried on April 2022 to identify BioProjects that met the following criteria: contained MTBC genomic sequences, could be linked to a published study, included genomes of clinical origin, multiple isolates per host were sequenced, and host IDs were available and could be linked to isolate IDs. Isolate IDs (run accession, BioSample accession, sample name) and basic metadata (patient ID, isolation source, collection date and geographical origin) were extracted from published studies, the NCBI BioSample and TB Portals.

***Genomic QC metrics and thresholds***

Given the diversity of studies genomic data was identified from, we assessed a variety of genomic QC metrics and applied stringent QC thresholds to discard cross-contamination with non-*MTBC* DNA, mixed infections of multiple *MTBC* strains, and to ensure clonality of isolates from the same host (see Methods and Supplementary Figure 1). This stringent QC was needed to minimize the rates of calling false-positive variants,^1^ a problem amplified when comparing highly related clonal genomes from the same strain. For defining genome QC thresholds, we considered all MTBC genomes available from the identified BioProjects, regardless of whether linked isolate-level metadata could be extracted or not. From Kraken2 reports the proportion of MTBC reads and that of the most abundant contaminating species was extracted. From TB-Profiler output, the presence of more than one *M. tuberculosis* sub-lineage (at the lowest sub-lineage level) was used as evidence of mixed infections. From Snippy derived *bam* and *vcf* files, we extracted the number of heterozygous sites and average sequencing depth. From QUAST reports we considered the total number of contigs, the total number of bases in the assembly and N50. We plotted the values of each of these metrics for all genomes to identify clear outliers and thresholds separating them form the rest of genomes (Supplementary Figure 1). Low-quality genomes were excluded from further analysis by applying the following thresholds: Kraken proportion of *M. tuberculosis* complex reads < 90%, average sequencing depth < 20%, percentage of missing call > 10%, heterozygous sites ratio > 0.002, number of contigs > 1,000, N50 < 150,000 bp and total assembly length < 4,0 Mb or > 5,5 Mb.

**Details of bioinformatics pipeline to identify *de novo* genetic variants**

The nucleotide sequence of the most recent common ancestor (MRCA) of all isolates from the same host was reconstructed with PastML v1.9.20^2^ from within-host phylogenies built with *RAxML* v8.2.8^3^, rooted on a closely related contextual isolate sampled from a different host (outgroup). This pipeline was implemented in four python scripts (identify_host_ancestral_isolate.step1.py to identify_host_ancestral_isolate.step4.py) available at <https://github.com/francesccoll/mtbc-adaptive-mutations/>. We excluded certain regions of the MTBC genome to minimize the rates of calling false-positive variants and inflating mutation counts. Variants in repetitive regions, detected by running Blastn v2.8.1+ on the reference genome against itself, and variants in regions of low complexity, as detected by *dustmasker*^4^ v1.0.0 using default settings, were also filtered out. We additionally masked regions defined as repetitive regions elsewhere and low confidence regions that are difficult to genotype using Illumina short reads.^5^ Overall, these masked regions accounted for a total of 735,968 bp (16.7% of the H37Rv reference genome).

***Source of protein structures***

The crystal structure of *M. tuberculosis* WhiB6 and σA4 complex was obtained from the PDB (PDB 8D5V). AlphaFold was used to generate 3D protein models for M. tuberculosis EccE_1_, EspK and MycP_1_. The AlphaFold model of *M. tuberculosis* MycP_1_ showed high structural conservation with the *M. smegmatis* orthologue (PDB 4KPG)^6^ with an r.m.s.d. of 0.299 Å over 312 Cα atoms, supporting the use of this model to map the mutations identified in this study. The structural model of the *M. tuberculosis* PhoR homodimer was predicted by AlphaFold. The dimerization-phosphorylation domain of PhoR was obtained from its crystal structure (PDB 5UKV).

**Electrostatic surface potential calculations**

Structural models for all proteins were obtained from AlphaFold^7^ predictions. Point mutations were introduced in silico using PyMOL (version 3.1.6.1 Schrödinger, LLC). Charge and radius parameters were assigned using PDB2PQR^8^ with the AMBER force field, and electrostatic surface potentials were calculated by solving the linearized Poisson–Boltzmann equation using APBS under default solvent conditions. Electrostatic potential maps were visualized and rendered in PyMOL using a consistent color scale to enable direct comparison between wild-type and mutant structures. Analyses were performed comparatively to evaluate relative electrostatic perturbations induced by each substitution.

**Protein stability predictions**

The potential impact of each mutation on protein stability was assessed using FoldX v5.1^9^ (BuildModel mode). Prior to mutation modeling, structures were optimized using the RepairPDB function. For each variant, five independent models were generated, and the average change in folding free energy (ΔΔG = G_mutant − G_wild-type) was calculated. Predicted ΔΔG values were interpreted as indicators of relative changes in thermodynamic stability. Because these analyses were performed on AlphaFold-derived models and in silico generated mutants, the results were considered qualitative estimates of stability trends rather than absolute quantitative measurements.

**Supplementary Results**

***Details of the compiled dataset of MTBC genomes***

In terms of geographical representation, *MTBC* strains originated from 27 different countries (Figure 1A), mostly sampled from Europe (578, 55.4%) and Africa (343, 32.9%). In Europe, most TB patients were sampled from Belarus (175, 16.8%), the United Kingdom (159, 15.2%), Moldova (114, 10.9%), and Georgia (76, 7.3%); and in Africa mostly from South Africa (276, 26.4%). As expected, most *MTBC* strains belonged to lineage 4 (519 out of 1,044, 49.7%) and 2 (439, 42.0%), while the rest were assigned to lineage 3 (50, 4.8%), 1 (33, 3.2%) and 5 (3, 0.3%) (Figure 1B). Most patients had isolates grown from pulmonary specimens (578, 55.4%), most often from sputum specimens only (526, 50.4%), followed by both sputum and bronchoalveolar lavages (16, 1.5%). Only 1 patient had extra-pulmonary specimens only, and 56 had both pulmonary and extra-pulmonary specimens. The anatomical origin of samples was not reported for 408 patients (39.1%).

In terms of baseline genotypic DR, there was an even and sizable representation of pan-susceptible (370, 35.4%), MDR (296, 28.4%), and pre-XDR strains (264, 25.3%). Among the other DR patterns (114, 10.9%), strains were mostly mono resistant to either isoniazid (49, 4.7%) or rifampicin (36, 3.4%) (Figure 1C). In terms of timing of collection between samples, these were available for most patients (699, 67%), of which 62 (5.9%) had samples collected on the same day, 366 (35.1%) within one month, 164 (15.7%) between one to twelve months, and 107 (10.3%) within more than one year apart (Figure 1D). For patients with unknown sampling dates (345, 33%), 139 (13.3%) patients had their samples collected within one year, 66 (6.3%) within two years, and the rest (58, 5.5%) within more than two years apart. The remaining 82 patients (7.9%) had no available information on sampling collection dates.

***Putative effect of protein-altering mutations in ESX-1 loci***

We identified eight PhoR mutations across the full-length protein (Figure 3A) including a substitution on the sensor domain (Arg80Met); another in the HAMP domain (Ala221Val), responsible for signal transduction from the transmembrane to cytoplasmic catalytic regions; and three mutations clustered within the dimerization-phosphorylation domain (Pro264Leu, Tyr275Asp and Ala279Val), of which Pro264Leu could impair the conformational flexibility essential for transmitting conformational changes from the sensor domain through the transmembrane and cytoplasmic helices to regulate kinase activity.^10^ Finally, three additional mutations (Leu324Arg, Arg335Trp and Gly350Cys) were found in the ATPase domain which could interfere with ATP binding, domain stability, or interdomain coupling required for efficient kinase activity.

To further characterize the potential structural and physicochemical consequences of these substitutions, electrostatic surface potential analyses were performed for PhoR wild-type and its variants. Substitutions involving gain or loss of formally charged residues produced clear local electrostatic perturbations (Supplementary Figure 3). The Arg80Met and Arg335Trp variants resulted in loss of positively charged arginine residues, reducing local positive surface potential and promoting redistribution of the electrostatic field in these regions (Supplementary Figure 3A,G). The Tyr275Asp mutation introduced a negatively charged aspartate residue, generating a marked increase in local negative electrostatic potential (Supplementary Figure 3D), while the Leu324Arg substitution enhanced positive surface potential through the introduction of a charged side chain (Supplementary Figure 3F). In contrast, mutations that did not modify formal charge (Ala221Val, Pro264Leu, Ala279Val and Gly350Cys) did not produce substantial alterations in the electrostatic landscape (Supplementary Figure 3B,C,E,H).

To determine whether these variants could affect predicted structural stability, FoldX analyses were performed (Supplementary Table 4). Several variants showed minimal or modest impact on predicted stability (Arg80Met, Ala221Val, Ala279Val and Leu324Arg; ΔΔG ≤ ~1.4 kcal/mol), indicating structural tolerance despite electrostatic alterations in some cases (e.g., Arg80Met and Leu324Arg). Other substitutions exhibited more pronounced destabilization. Pro264Leu and Arg335Trp displayed moderate destabilizing effects (ΔΔG ≈ 3.5 and 3.2 kcal/mol, respectively), independent of major electrostatic changes in the case of Pro264Leu. The Tyr275Asp mutation showed strong predicted destabilization (ΔΔG ≈ 7.1 kcal/mol), consistent with the introduction of a negatively charged residue and a marked increase in ΔΔG. Notably, Gly350Cys presented the highest destabilizing effect among PhoR variants (ΔΔG ≈ 14.1 kcal/mol) despite lacking major electrostatic alterations, indicating that structural constraints at this position, rather than electrostatic factors, likely underlie the predicted instability.

Figure 3B shows that WhiB6 substitutions (Thr51Pro, detected twice, and Arg64His) are not located within the regions predicted to interact with DNA^11^ or with the RNA polymerase α-subunit^12^ but within α-helices that contain conserved cysteines coordinating the [4Fe-4S] cluster. Interestingly, a recent study^13^ found 13 independent WhiB6 variants linked to decreases in PE35-PPE68-esxBA operon expression, including substitutions in WhiB6 close to the ones detected here (Cys53Gly, Asp46Ala). Electrostatic analyses of WhiB6 variants showed that Arg64His reduced local positive potential, allowing a neighboring aspartate residue to dominate the electrostatic environment (Supplementary Figure 4B). Thr51Pro removed the threonine hydroxyl group, resulting in subtle local redistribution without affecting the global electrostatic profile (Supplementary Figure 4A). FoldX predicted both variants to be destabilizing (Supplementary Table 4), with Arg64His showing stronger effects (ΔΔG ≈ 4.5 kcal/mol) than Thr51Pro (ΔΔG ≈ 3.1 kcal/mol), consistent with localized structural perturbation rather than global unfolding.

EspK is an auxiliary protein that recruits ESX-1 substrate EspB through its C-terminal domain, while its N-terminal domain interacts with the core secretion component EccCb_1_ to guide EspB towards the secretion pore.^14,15^ We identified five mutations in EspK (Figure 3C): two missense variants (Leu39Trp and Ile262Thr) and three frameshifts (Gln149fs, Ala223fs, and Ala366fs), the latter predicted to produce truncated and non-functional EspK that would compromise EspB secretion. For EspK, neither missense mutation substantially altered surface electrostatics.The Leu39Trp substitution, located in a buried region of the protein, did not produce detectable changes in surface electrostatics, consistent with the absence of formal charge alteration (Supplementary Figure 4C). Similarly, the Ile262Thr variant, introduced a polar but uncharged residue and did not substantially modify the electrostatic landscape (Supplementary Figure 4D). FoldX analysis (Supplementary Table 4) predicted Ile262Thr to be structurally neutral (ΔΔG ≈ −0.07 kcal/mol), while Leu39Trp exhibited strong predicted destabilization (ΔΔG ≈ 11.6 kcal/mol), largely driven by van der Waals clashes.

In MycP_1_ we identified three missense mutations (Gly112Asp, Ala175Gly, and Gly280Ser), the first two within or adjacent to the α-helices forming the substrate-binding pocket but distant from the catalytic triad (Figure 3D), thus not expected to directly affect proteolytic activity but might influence substrate recognition or enzyme dynamics. Among the three missense variants, only Gly112Asp introduced a formally charged residue, resulting in a pronounced local increase in negative electrostatic potential at this position (Supplementary Figure 4E). In contrast, Ala175Gly and Gly280Ser did not produce detectable electrostatic alterations (Supplementary Figure 4F-G). FoldX (Supplementary Table 4) predicted strong destabilization for Gly112Asp (ΔΔG ≈ 8.6 kcal/mol), consistent with the introduction of a negatively charged side chain and structural constraints within the substrate-binding region. Gly280Ser showed moderate-to-strong destabilization (ΔΔG ≈ 4.2 kcal/mol), primarily driven by steric contributions, despite minimal electrostatic alteration. In contrast, Ala175Gly displayed only mild predicted destabilization (ΔΔG ≈ 1.4 kcal/mol), suggesting relative structural tolerance.

In EccE_1_ we identified four mutations: two missense substitutions (Thr174Pro and Pro413Thr) in surface-exposed loops of the cytosolic domain (Figure 3E), where they could affect local flexibility or protein-protein interactions; and two frameshift variants (Pro290fs and Pro294fs) predicted to generate truncated proteins. Electrostatic mapping revealed only subtle local redistribution for Thr174Pro and Pro413Thr (Supplementary Figure 4H,I). Consistently FoldX predictions indicated limited impact on overall stability for EccE_1_ missense variants (Supplementary Table 4).

**Supplementary Table 1.** Number of MTBC isolate genomes and TB patients available per study before and after genomic QC

|  | Before genomic QC | | | | After genomic QC | | | | | |
| --- | --- | --- | --- | --- | --- | --- | --- | --- | --- | --- |
|  | N isolates | | | N hosts | N isolates | | | | | N hosts |
| Study ID (citation) | total | context | within host | total | removed | total | context | same  strain | different  strain | kept |
| TB portals^16^ | 3,211 | 2,355 | 856 | 383 | 104 | 3,107 | 2,298 | 630 | 179 | 276 |
| cox2021^17^ | 1,410 | 1,174 | 236 | 118 | 40 | 1,370 | 1,146 | 192 | 32 | 96 |
| nimmo2020^18^ | 615 | 246 | 369 | 118 | 107 | 508 | 229 | 277 | 2 | 95 |
| wollenberg2017^19^ | 447 | 0 | 447 | 97 | 26 | 421 | 1 | 409 | 11 | 95 |
| walker2013^20^ | 388 | 166 | 222 | 86 | 8 | 380 | 159 | 210 | 11 | 81 |
| walker2018b^21^ | 1,555 | 1,338 | 217 | 89 | 54 | 1,501 | 1,292 | 200 | 9 | 81 |
| lieberman2016^22^ | 2,693 | 0 | 2,693 | 44 | 214 | 2,479 | 0 | 2,478 | 1 | 44 |
| guerra2014^23^ | 168 | 0 | 168 | 84 | 39 | 129 | 31 | 64 | 34 | 32 |
| chen2021^24^ | 83 | 0 | 83 | 31 | 13 | 70 | 3 | 66 | 1 | 28 |
| perez-lago2021^25^ | 68 | 0 | 68 | 27 | 5 | 63 | 2 | 61 | 0 | 25 |
| dippenaar2019^26^ | 50 | 0 | 50 | 25 | 3 | 47 | 3 | 42 | 2 | 21 |
| asare2021^27^ | 60 | 0 | 60 | 29 | 0 | 60 | 0 | 42 | 18 | 20 |
| casali2016^28^ | 38 | 0 | 38 | 19 | 0 | 38 | 0 | 38 | 0 | 19 |
| witney2017^29^ | 69 | 1 | 68 | 34 | 21 | 48 | 12 | 36 | 0 | 18 |
| liu2020^30^ | 804 | 0 | 804 | 18 | 40 | 764 | 0 | 764 | 0 | 18 |
| folkvardsen2020^31^ | 82 | 0 | 82 | 32 | 29 | 53 | 11 | 38 | 4 | 17 |
| shanmugam2021^32^ | 82 | 0 | 82 | 41 | 25 | 57 | 17 | 28 | 12 | 14 |
| chen2020^33^ | 151 | 102 | 49 | 21 | 20 | 131 | 90 | 34 | 7 | 14 |
| ssengooba2016b^34^ | 26 | 0 | 26 | 13 | 2 | 24 | 2 | 22 | 0 | 11 |
| hu2021^35^ | 130 | 0 | 130 | 63 | 31 | 99 | 19 | 22 | 58 | 11 |
| clark2013^36^ | 51 | 36 | 15 | 5 | 0 | 51 | 36 | 13 | 2 | 4 |
| mokrousov2020^37^ | 20 | 0 | 20 | 6 | 4 | 16 | 2 | 14 | 0 | 4 |
| acosta2022^38^ | 33 | 1 | 32 | 3 | 3 | 30 | 1 | 29 | 0 | 3 |
| seraphin2019^39^ | 31 | 0 | 31 | 3 | 0 | 31 | 0 | 31 | 0 | 3 |
| xu2018^40^ | 18 | 0 | 18 | 4 | 3 | 15 | 0 | 12 | 3 | 3 |
| mehaffy2014^41^ | 66 | 60 | 6 | 3 | 19 | 47 | 43 | 4 | 0 | 2 |
| merker2013^42^ | 13 | 0 | 13 | 4 | 3 | 10 | 1 | 7 | 2 | 2 |
| wada2015^43^ | 10 | 8 | 2 | 1 | 0 | 10 | 8 | 2 | 0 | 1 |
| cancino2019^44^ | 16 | 0 | 16 | 1 | 0 | 16 | 0 | 15 | 1 | 1 |
| eldholm2014^45^ | 18 | 0 | 18 | 1 | 0 | 18 | 0 | 18 | 0 | 1 |
| ley2021^46^ | 12 | 10 | 2 | 1 | 1 | 11 | 9 | 2 | 0 | 1 |
| black2015^47^ | 4 | 0 | 4 | 2 | 1 | 3 | 1 | 2 | 0 | 1 |
| hjort2021^48^ | 77 | 0 | 77 | 1 | 1 | 76 | 0 | 76 | 0 | 1 |
| martin2018^49^ | 25 | 21 | 4 | 1 | 20 | 5 | 1 | 4 | 0 | 1 |
| liu2015^50^ | 5 | 0 | 5 | 1 | 5 | 0 | 0 | 0 | 0 | 0 |
|  | | | | | | | | | | |
| Total | 12,529 | 5,518 | 7,011 | 1,409 | 841 | 11,688 | 5,417 | 5,882 | 389 | 1,044 |

The “within host” isolate genomes are those taken from TB patients who had multiple isolates sequenced per host. The total number of these patients per study is indicated in the fifth column (N hosts total). In addition, isolate genomes from other patients who had a single MTBC genome sequenced per host were considered as contextual isolates (“context”). The total number of isolates before genomic QC corresponds to the sum of “within host” and “context” isolates. After genomic QC, the genomes that did not meet the genomic QC criteria were discarded (“removed”). The “within host” isolate genomes were split between those assigned to a different strain (“different strain”) and those assigned to the same strain (“same strain”), which were kept for further analyses. The number of patients with multiple isolates sequenced of the same strain is indicated in “N hosts kept” (total 1,044 patients across studies).

**Supplementary Table 2** *M. tuberculosis* genes known to confer resistance to anti-tuberculosis drugs

| **Drug** | **Line** | **Locus tag (gene name)*** |
| --- | --- | --- |
|  |  |  |
| Amikacin | 2^nd^ | Rv0529 (*ccsA*), **Rv2416c (*eis*) promoter**, Rv3106 (*fprA*), **MTB000019 (*rrs*)**, Rv3805c (*aftB*), Rv3862c (*whiB6*), Rv3197A (*whiB7*) |
| Bedaquiline | last | Rv1305 (*atpE*), Rv2535c (*pepQ*), Rv1979c, **Rv0678**, Rv0676c (*mmpL5*), Rv0677c (*mmpS5*) |
| Capreomycin | 2^nd^ | Rv0529 (*ccsA*), Rv3106 (*fprA*), **MTB000019 (*rrs*)**, **Rv1694 (*tlyA*)**, Rv3805c (*aftB*), Rv3862c (*whiB6*) |
| Clofazimine | last | Rv2535c (*pepQ*), Rv1979c, **Rv0678**, Rv0676c (*mmpL5*), Rv0677 (*mmpS5*) |
| Cycloserine | last | **Rv2780 (*ald*)**, **Rv3423c (*alr*)** |
| Delamanid | last | Rv3547 (ddn), Rv3261- Rv3262 (fbiA-fbiB), Rv1173 (fbiC), Rv2983, Rv0407 (fgd1) |
| Ethambutol | 1^st^ | **Rv3795 (*embB*)**, Rv3793 (*embC*), Rv3794 (*embA*), Rv3792 (*aftA*), Rv1267c (*embR*), Rv3806c (*ubiA*) |
| Ethionamide | 2^nd^ | Rv3083, Rv1484 (*inhA*), Rv0486 (*mshA*), Rv1854c (*ndh*), **Rv3854c (*ethA*)**, **Rv3854c (*ethA*) promoter,** Rv3855 (*ethR*), Rv0565c |
| Isoniazid | 1^st^ | **Rv1908 (*katG*)**, Rv2428 (*aphC*), **Rv2428 (*aphC*) promoter**, Rv1484 (*inhA*), **Rv1484 (*inhA*) promoter**, Rv0486 (*mshA*), Rv1854c (*ndh*), Rv1258c, Rv2752c |
| Kanamycin | 2^nd^ | **MTB000019 (*rrs*)**, **Rv2416c (*eis*) promoter**, Rv3197A (*whiB7*) |
| Fluoroquinolones | 2^nd^ | **Rv0006 (*gyrA*), Rv0005 (*gyrB*)** |
| Linezolid | last | Rv0701 (*rplC*), MTB000020 (*rrl*) |
| PAS** | last | **Rv2764c (*thyA*)**^51^, **Rv2754c (*thyX*)**^52^ **promoter** |
| Pyrazinamide | 1^st^ | Rv3596c (*clpC1*), Rv3601 (*panD*), **Rv2043c (*pncA*)**, Rv1918c (*PPE35*), Rv1258c, Rv3236c |
| Rifampicin | 1^st^ | **Rv0667 (*rpoB*)**, **Rv0957 (*rpoC*)**, Rv3457c (*rpoA*), Rv2752c |
| Streptomycin | 2^nd^ | **Rv3919c (*gid*)**, **Rv0682 (*rpsL*)**, **MTB000019 (*rrs*),** Rv1258c Rv3862c (*whiB6*), Rv3197A (*whiB7*) |

*** Whole list of genes extracted from. In bold, drug resistance genes detected in this study with signals of *in host* mutational adaption. Genes approaching statistical significance in this analysis are underscored. **PAS, para-aminosalicylic acid. Genes linked to PAS resistance not included in^16^ but identified from other sources.^51,52^

**Supplementary Table 3.** Data sources and treatment data availability.

| **Study ID (citation)** | **Number of strains** | **Treatment data** |
| --- | --- | --- |
| acosta2022^38^ | 3 | *Not available* |
| asare2021^27^ | 20 | Available |
| black2015^47^ | 1 | *Not available* |
| cancino2019^44^ | 1 | Available |
| casali2016^28^ | 19 | *Not available* |
| chen2020^33^ | 14 | *Not available* |
| chen2021^24^ | 28 | *Not available* |
| clark2013^36^ | 5 | Available |
| cox2021^17^ | 96 | *Not available* |
| dippenaar2019^26^ | 21 | *Not available* |
| eldholm2014^45^ | 1 | *Not available* |
| folkvardsen2020^31^ | 12 | *Not available* |
| guerra2014^23^ | 32 | Available |
| hjort2021^48^ | 1 | Available |
| hu2021^35^ | 11 | Available |
| ley2021^46^ | 1 | *Not available* |
| lieberman2016^22^ | 45 | *Not available* |
| liu2020^30^ | 19 | *Not available* |
| martin2018^49^ | 1 | *Not available* |
| mehaffy2014^41^ | 12 | *Not available* |
| merker2013^42^ | 2 | *Not available* |
| mokrousov2020^37^ | 4 | Available |
| nimmo2020^18^ | 95 | Available |
| perez-lago2021^25^ | 25 | *Not available* |
| seraphin2019^39^ | 3 | *Not available* |
| shanmugam2021^32^ | 14 | *Not available* |
| ssengooba2016b^34^ | 11 | *Not available* |
| TB Portals^16^ | 285 | Available |
| wada2015^43^ | 1 | *Not available* |
| walker2013^20^ | 81 | *Not available* |
| walker2018b^21^ | 81 | *Not available* |
| witney2017^29^ | 18 | Available |
| wollenberg2017^19^ | 99 | Available |
| xu2018^40^ | 3 | Available |

# **Supplementary Table 4**. FoldX Stability Predictions and Electrostatic Alterations

| **Protein** | **Mutation** | **Mean ΔΔG± SD (kcal/mol)^A^** | **Predicted effect^B^** | **Main contributing term^C^** | **Electrostatic potential alteration^D^** |
| --- | --- | --- | --- | --- | --- |
| PhoR | R80M | −0.28 ± 1.00 | Neutral | — | Yes (loss of +charge).  See Fig. S3A |
| PhoR | A221V | 1.39 ± 0.25 | Mild destabilizing | — | No  See Fig. S3B |
| PhoR | P264L | 3.54 ± 1.14 | Strong destabilizing | Solvation Polar | No  See Fig. S3C |
| PhoR | Y275D | 7.11 ± 0.29 | Strong destabilizing | Solvation Hydrophobic | Yes (gain of −charge). See Fig. S3D |
| PhoR | A279V | 0.92 ± 0.09 | Neutral | — | No. See Fig. S3E |
| PhoR | L324R | 1.18 ± 0.14 | Mild destabilizing | — | Yes (gain of +charge). See Fig. S3F |
| PhoR | R335W | 3.16 ± 0.21 | Strong destabilizing | Entropy sidechain | Yes (loss of +charge). See Fig. S3G |
| PhoR | G350C | 14.13 ± 0.65 | Strong destabilizing | Van der Waals clashes | No  See Fig. S3H |
| WhiB6 | T51P | 3.07 ± 0.06 | Strong destabilizing | Van der Waals clashes | Redistribution (no formal charge change). See Fig. S4A |
| WhiB6 | R64H | 4.53 ± 1.17 | Strong destabilizing | Electrostatics/ Van der Waals clashes | Yes (loss of +charge).  See Fig. S4B |
| EspK | L39W | 11.59 ± 0.60 | Strong destabilizing | Van der Waals clashes | No  See Fig. S4C |
| EspK | I262T | −0.07 ± 0.00 | Neutral | — | No. See Fig. S4D |
| MycP1 | G112D | 8.58 ± 0.84 | Strong destabilizing | Van der Waals clashes | Yes (gain of −charge). See Fig. S4E |
| MycP1 | A175G | 1.38 ± 0.00 | Mild destabilizing | — | No  See Fig. S4F |
| MycP1 | G280S | 4.22 ± 0.93 | Strong destabilizing | Van der Waals clashes | No  See Fig. S4G |
| EccE1 | T174P | −0.17 ± 0.00 | Neutral | — | Redistribution (loss of OH). See Fig. S4H |
| EccE1 | P413T | 1.41 ± 0.01 | Mild destabilizing | — | Redistribution (gain of OH). See Fig. S4I |

(A) Mean ΔΔG values (kcal/mol) calculated using FoldX (BuildModel mode) based on AlphaFold-predicted structural models represent the difference in total folding energy between the mutant and the corresponding wild-type protein (ΔΔG = G_mutant − G_wild-type). Values are shown as mean ± standard deviation from five independent models.

(B) Predicted effect classification based on ΔΔG magnitude: neutral (<1 kcal/mol), mild destabilizing (1–2 kcal/mol), moderate destabilizing (2–3 kcal/mol), or strong destabilizing (>3 kcal/mol).
(C) Main contributing term indicates the FoldX energy component with the largest contribution to destabilization in mutations with |ΔΔG| ≥ 3 kcal/mol.

(D) Electrostatic potential alteration summarizes the qualitative impact of each mutation on surface electrostatic potential as determined by APBS calculations. See supplementary figure 3 and 4.

**Supplementary Figure 1** Visual representation of genomic QC metrics and thresholds

The scatter plots of individual genomic QC metrics are shown as different panels: (A) Average sequencing depth, (B) heterozygous call ratio, (C) missing base call percentage, (D) Kraken percentage of MTBC reads, (E) assembly N50, (F) number of contigs in assembly, and (G) total length of assembled genome. The values for each QC metric were plotted in a scatter plot to visually identify outliers. In most cases, a cut-off line can be drawn to easily separate outliers from the rest and set QC thresholds. For average sequencing depth, a cut-off of 20 was used in this study.

**Supplementary Figure 2.** Descriptive characteristics of all strains with available drug treatment data.

**Other resistant* refers to any strains that had predicted resistance to more than one drug but did not meet the clinical MDR or XDR definitions.

**Supplementary Figure 3.** Electrostatic surface potential of PhoR wild-type and variant proteins.

Electrostatic surface potentials were calculated using the Adaptive Poisson–Boltzmann Solver (APBS) at pH 7.0 based on AlphaFold-predicted structural models. Potentials are mapped onto the molecular surface using a uniform color scale ranging from −5 kT/e (red) to +5 kT/e (blue), as indicated by the spectrum bar. Values below −5 kT/e and above +5 kT/e are shown in saturated red and blue, respectively. All structures are displayed in the same orientation to enable direct comparison between wild-type and mutant proteins. PhoR wild-type and R80M (A), A221V (B), P264L (C), Y275D (D), A279V (E), L324R (F), R335W (G), and G350C (H) variants are shown.

**Supplementary Figure 4.** Electrostatic surface potential of WhiB6, EspK, MycP1 and Ecc1 wild-type and variant proteins.

Electrostatic surface potentials were calculated using APBS at pH 7.0 based on AlphaFold-predicted structural models and visualized using a uniform color scale from −5 kT/e (red) to +5 kT/e (blue). All structures are shown in the same orientation to allow direct comparison. Wild-type and T51P (A) and R64H (B) for WhiB6; L39W (C) and I262T (D) for EspK; G112D (E), A175G (F), and G280S (G) for MycP1; and T174P (H) and P413T (I) for Ecc1 are displayed.

**Supplementary References**

1.	Vargas, R. *et al.* In-host population dynamics of Mycobacterium tuberculosis complex during active disease. *eLife* **10**, e61805 (2021).

2.	Ishikawa, S. A., Zhukova, A., Iwasaki, W. & Gascuel, O. A Fast Likelihood Method to Reconstruct and Visualize Ancestral Scenarios. *Mol. Biol. Evol.* **36**, 2069–2085 (2019).

3.	Stamatakis, A. RAxML version 8: a tool for phylogenetic analysis and post-analysis of large phylogenies. *Bioinformatics* **30**, 1312–1313 (2014).

4.	Morgulis, A., Gertz, E. M., Schäffer, A. A. & Agarwala, R. A Fast and Symmetric DUST Implementation to Mask Low-Complexity DNA Sequences. *J. Comput. Biol.* **13**, 1028–1040 (2006).

5.	Marin, M. *et al.* Benchmarking the empirical accuracy of short-read sequencing across the M. tuberculosis genome. *Bioinformatics* **38**, 1781–1787 (2022).

6.	Solomonson, M. *et al.* Structure of the Mycosin-1 Protease from the Mycobacterial ESX-1 Protein Type VII Secretion System. *J. Biol. Chem.* **288**, 17782–17790 (2013).

7.	Abramson, J. *et al.* Accurate structure prediction of biomolecular interactions with AlphaFold 3. *Nature* **630**, 493–500 (2024).

8.	Jurrus, E. *et al.* Improvements to the APBS biomolecular solvation software suite. *Protein Sci.* **27**, 112–128 (2018).

9.	Schymkowitz, J. *et al.* The FoldX web server: an online force field. *Nucleic Acids Res.* **33**, W382–W388 (2005).

10.	Xing, D. *et al.* Asymmetric Structure of the Dimerization Domain of PhoR, a Sensor Kinase Important for the Virulence of *Mycobacterium tuberculosis*. *ACS Omega* **2**, 3509–3517 (2017).

11.	Teena, Kumari, S., Singh, R., Siddhanta, S. & Deep, S. Biophysical characterization and interaction study of WhiB6 protein of *Mycobacterium tuberculosis* with *espA* . *FEBS J.* febs.70202 (2025) doi:10.1111/febs.70202.

12.	Feng, L., Chen, Z., Wang, Z., Hu, Y. & Chen, S. Genome-wide characterization of monomeric transcriptional regulators in Mycobacterium tuberculosis. *Microbiology* **162**, 889–897 (2016).

13.	Culviner, P. H. *et al.* Evolution of Mycobacterium tuberculosis transcription regulation is associated with increased transmission and drug resistance. *Cell* S0092867425010360 (2025) doi:10.1016/j.cell.2025.09.005.

14.	Gijsbers, A. *et al.* The crystal structure of the EspB-EspK virulence factor-chaperone complex suggests an additional type VII secretion mechanism in Mycobacterium tuberculosis. *J. Biol. Chem.* **299**, 102761 (2023).

15.	Lim, Z. L., Drever, K., Dhar, N., Cole, S. T. & Chen, J. M. Mycobacterium tuberculosis EspK Has Active but Distinct Roles in the Secretion of EsxA and EspB. *J. Bacteriol.* **204**, e00060-22 (2022).

16.	Rosenthal, A. *et al.* The TB Portals: an Open-Access, Web-Based Platform for Global Drug-Resistant-Tuberculosis Data Sharing and Analysis. *J. Clin. Microbiol.* **55**, 3267–3282 (2017).

17.	Cox, H. *et al.* Potential contribution of HIV during first-line tuberculosis treatment to subsequent rifampicin-monoresistant tuberculosis and acquired tuberculosis drug resistance in South Africa: a retrospective molecular epidemiology study. *Lancet Microbe* **2**, e584–e593 (2021).

18.	Nimmo, C. *et al.* Dynamics of within-host Mycobacterium tuberculosis diversity and heteroresistance during treatment. *eBioMedicine* **55**, 102747 (2020).

19.	Wollenberg, K. R. *et al.* Whole-Genome Sequencing of Mycobacterium tuberculosis Provides Insight into the Evolution and Genetic Composition of Drug-Resistant Tuberculosis in Belarus. *J. Clin. Microbiol.* **55**, 457–469 (2017).

20.	Walker, T. M. *et al.* Whole-genome sequencing to delineate Mycobacterium tuberculosis outbreaks: a retrospective observational study. *Lancet Infect. Dis.* **13**, 137–46 (2013).

21.	Walker, A. S. & Peto, T. E. A. Prediction of Susceptibility to First-Line Tuberculosis Drugs by DNA Sequencing. *N. Engl. J. Med.* **379**, 1403–1415 (2018).

22.	Lieberman, T. D. *et al.* Genomic diversity in autopsy samples reveals within-host dissemination of HIV-associated Mycobacterium tuberculosis. *Nat. Med.* **22**, 1470–1474 (2016).

23.	Guerra-Assunção, J. A. *et al.* Recurrence due to Relapse or Reinfection With *Mycobacterium tuberculosis*: A Whole-Genome Sequencing Approach in a Large, Population-Based Cohort With a High HIV Infection Prevalence and Active Follow-up. *J. Infect. Dis.* **211**, 1154–1163 (2015).

24.	Chen, Y. *et al.* Lesion Heterogeneity and Long-Term Heteroresistance in Multidrug-Resistant Tuberculosis. *J. Infect. Dis.* **224**, 889–893 (2021).

25.	Pérez‐Lago, L. *et al.* Recurrences of multidrug‐resistant tuberculosis: Strains involved, within‐host diversity, and fine‐tuned allocation of reinfections. *Transbound. Emerg. Dis.* **69**, 327–336 (2022).

26.	Dippenaar, A. *et al.* Whole genome sequencing provides additional insights into recurrent tuberculosis classified as endogenous reactivation by IS6110 DNA fingerprinting. *Infect. Genet. Evol.* **75**, 103948 (2019).

27.	Asare, P. *et al.* Genomic epidemiological analysis identifies high relapse among individuals with recurring tuberculosis and provides evidence of recent household-related transmission of tuberculosis in Ghana. *Int. J. Infect. Dis.* **106**, 13–22 (2021).

28.	Casali, N. *et al.* Whole Genome Sequence Analysis of a Large Isoniazid-Resistant Tuberculosis Outbreak in London: A Retrospective Observational Study. *PLOS Med.* **13**, e1002137 (2016).

29.	Witney, A. A. *et al.* Use of whole-genome sequencing to distinguish relapse from reinfection in a completed tuberculosis clinical trial. *BMC Med.* **15**, 1–13 (2017).

30.	Liu, Q. *et al.* *Mycobacterium tuberculosis* clinical isolates carry mutational signatures of host immune environments. *Sci. Adv.* **6**, eaba4901 (2020).

31.	Folkvardsen, D. B. *et al.* Recurrent tuberculosis in patients infected with the predominant Mycobacterium tuberculosis outbreak strain in Denmark. New insights gained through whole genome sequencing. *Infect. Genet. Evol.* **80**, 104169 (2020).

32.	Shanmugam, S. *et al.* Whole genome sequencing based differentiation between re-infection and relapse in Indian patients with tuberculosis recurrence, with and without HIV co-infection. *Int. J. Infect. Dis.* **113**, S43–S47 (2021).

33.	Chen, X. *et al.* Analysis of Serial Multidrug-Resistant Tuberculosis Strains Causing Treatment Failure and Within-Host Evolution by Whole-Genome Sequencing. *mSphere* **5**, e00884-20 (2020).

34.	Ssengooba, W., de Jong, B. C., Joloba, M. L., Cobelens, F. G. & Meehan, C. J. Whole genome sequencing reveals mycobacterial microevolution among concurrent isolates from sputum and blood in HIV infected TB patients. *BMC Infect. Dis.* **16**, 1–7 (2016).

35.	Hu, Y. *et al.* Emergence of additional drug resistance during treatment of multidrug-resistant tuberculosis in China: a prospective cohort study. *Clin. Microbiol. Infect.* **27**, 1805–1813 (2021).

36.	Clark, T. G. *et al.* Elucidating Emergence and Transmission of Multidrug-Resistant Tuberculosis in Treatment Experienced Patients by Whole Genome Sequencing. *PLoS ONE* **8**, e83012 (2013).

37.	Mokrousov, I. *et al.* Frequent acquisition of bedaquiline resistance by epidemic extensively drug-resistant Mycobacterium tuberculosis strains in Russia during long-term treatment. *Clin. Microbiol. Infect.* **27**, 478–480 (2021).

38.	Acosta, F. *et al.* Insights into the Complexity of a Dormant *Mycobacterium tuberculosis* Cluster Once Transmission Is Resumed. *Microbiol. Spectr.* **10**, e01381-21 (2022).

39.	Séraphin, M. N. *et al.* Direct transmission of within-host Mycobacterium tuberculosis diversity to secondary cases can lead to variable between-host heterogeneity without de novo mutation: A genomic investigation. *EBioMedicine* **47**, 293–300 (2019).

40.	Xu, Y. *et al.* In vivo evolution of drug-resistant Mycobacterium tuberculosis in patients during long-term treatment. *BMC Genomics* **19**, 640 (2018).

41.	Mehaffy, C. *et al.* Marked Microevolution of a Unique Mycobacterium tuberculosis Strain in 17 Years of Ongoing Transmission in a High Risk Population. *PLoS ONE* **9**, e112928 (2014).

42.	Merker, M. *et al.* Whole genome sequencing reveals complex evolution patterns of multidrug-resistant Mycobacterium tuberculosis Beijing strains in patients. *PloS One* **8**, e82551 (2013).

43.	Wada, T. *et al.* Clonality and Micro-Diversity of a Nationwide Spreading Genotype of Mycobacterium tuberculosis in Japan. *Plos One* **10**, e0118495 (2015).

44.	Cancino-Muñoz, I. *et al.* Cryptic Resistance Mutations Associated With Misdiagnoses of Multidrug-Resistant Tuberculosis. *J. Infect. Dis.* **220**, 316–320 (2019).

45.	Eldholm, V. *et al.* Evolution of extensively drug-resistant Mycobacterium tuberculosisfrom a susceptible ancestor in a single patient. *Genome Biol.* **15**, 490 (2014).

46.	Ley, S. D. *et al.* Melting the *eis* : Nondetection of Kanamycin Resistance Markers by Routine Diagnostic Tests and Identification of New *eis* Promoter Variants. *Antimicrob. Agents Chemother.* **65**, e02502-20 (2021).

47.	Black, P. *et al.* Whole genome sequencing reveals genomic heterogeneity and antibiotic purification in Mycobacterium tuberculosis isolates. *BMC Genomics* **16**, 857 (2015).

48.	Hjort, K. *et al.* Dynamics of Extensive Drug Resistance Evolution of *Mycobacterium tuberculosis* in a Single Patient During 9 Years of Disease and Treatment. *J. Infect. Dis.* **225**, 1011–1020 (2022).

49.	Martin, M. A., Lee, R. S., Cowley, L. A., Gardy, J. L. & Hanage, W. P. Within-host Mycobacterium tuberculosis diversity and its utility for inferences of transmission. *Microb. Genomics* **4**, (2018).

50.	Liu, Q. *et al.* Within patient microevolution of Mycobacterium tuberculosis correlates with heterogeneous responses to treatment. *Sci. Rep.* **5**, 17507 (2015).

51.	Pandey, B., Grover, S., Kaur, J. & Grover, A. Analysis of mutations leading to para-aminosalicylic acid resistance in Mycobacterium tuberculosis. *Sci. Rep.* **9**, 13617 (2019).

52.	Zeng, R. *et al.* Association between mutations in a thyX-hsdS.1 region and para -aminosalicylic acid resistance in Mycobacterium tuberculosis clinical isolates. *Emerg. Microbes Infect.* **12**, (2023).
